# Cavitation Erosion of Cermet-Coated Aluminium Bronzes

**DOI:** 10.3390/ma9030204

**Published:** 2016-03-17

**Authors:** Ion Mitelea, Octavian Oancă, Ilare Bordeaşu, Corneliu M. Crăciunescu

**Affiliations:** 1 Department of Materials and Manufacturing Engineering, Politehnica University of Timisoara, 300026 Timisoara, Romania; craciunescucm@yahoo.com; 2 Department of Mechanical Machines, Equipments and Transportation, Politehnica University of Timisoara, 300026 Timisoara, Romania; octavian.oanca@yahoo.com (O.O.); ilare.bordeasu@upt.ro (I.B.)

**Keywords:** cavitation erosion, bronze-alloys, plasma spray, oxide, metal, laser remelting

## Abstract

The cavitation erosion resistance of CuAl10Ni5Fe2.5Mn1 following plasma spraying with Al_2_O_3_·30(Ni_20_Al) powder and laser re-melting was analyzed in view of possible improvements of the lifetime of components used in hydraulic environments. The cavitation erosion resistance was substantially improved compared with the one of the base material. The thickness of the re-melted layer was in the range of several hundred micrometers, with a surface microhardness increasing from 250 to 420 HV 0.2. Compositional, structural, and microstructural explorations showed that the microstructure of the re-melted and homogenized layer, consisting of a cubic Al_2_O_3_ matrix with dispersed Ni-based solid solution is associated with the hardness increase and consequently with the improvement of the cavitation erosion resistance.

## 1. Introduction

The cavitation erosion is a complex phenomenon of interactions of hydrostatic, mechanical, metallurgical, and chemical processes. According to Brennen [1] the cavitation damage is produced by repetitive stresses that occur when bubbles collapse on the surface, thus leading to local cracking and fatigue failure. Franc and Michel [2] observed that, besides micro bubble collapse, several additional phenomena contribute to the cavitation erosion process: an impinging microjet, collective microbubble cloud collapse, and impacting cavitating vortices.

The cavitation erosion can lead to significant losses of material localized in the surface area of components used in hydraulic equipment, which can cause major failures in service and degradation. Its study under laboratory conditions can be useful in redesigning components, to improve the selection of materials or to establish new technologies that would provide improved cavitation erosion resistance. Aperador *et al.* [3] recently used this laboratory approach to study the erosion corrosion resistance of aluminum exposed to bioethanol mixtures by TiN coating. The cavitation erosion can be reduced by changing the hydrodynamic conditions at the interface between the cavitation fluid and the component by modifying the properties of the surface of the materials in contact with the fluid, as remarked by Hattori and T. Kitagawa [4] in their analysis of cavitation erosion resistance of cast iron and nonferrous metals based on databases and comparisons with carbon steel data.

Copper-based alloys, such as brass or bronzes, are considered for the fabrication of components used in fluid environments-with the advantage of convenient casting and machining technologies. However, differences exist between the two classes of alloys concerning the cavitation erosion behavior. Yu *et al.* [5] analyzed the cavitation erosion corrosion behavior of manganese-nickel-aluminum bronze in comparison to manganese-brass and found that the cumulative mass loss of a manganese bronze was about 1/3 of the one of the manganese-brass. They attributed the difference to its lower stacking fault energy, higher microhardness and work-hardening ability, and to a favorable propagation of cavitation cracks, *i.e.*, parallel to the surface-for the bronze, compared to the propagation perpendicular to the surface for the brass.

Several bronze alloys were developed over time, to be used in the petrochemical industry, as well as for casting pump components and ship propellers. Based on a literature survey, Couture [6] showed that—for propellers—bronzes have very good mechanical properties, excellent fatigue strength, as well as good wear resistance and cavitation erosion behavior in seawater. Since they are used for manufacturing components designed to operate in hydraulic environments, the cavitation erosion behavior and the behavior in corrosive environments of aluminum bronzes is significant. In an early study on face centred cubic (f.c.c.) metals, Suh and Saka [7] concluded that the delamination wear (*i.e.*, surface traction, plastic strain accumulation, crack nucleation, and crack propagation) is a too complex a process to be described by a simple relationship between the stacking fault energy and the wear rate. On the other hand, Reddy *et al.* [8] analyzed the effect of stacking fault energy on solid particle erosion-based on a series of copper alloys, and found that the erosion rate correlates well to the stacking fault energy and the strain hardening exponent. The observation was further confirmed by the experiments of Zhang and Fang [9], with studies on the cavitation erosion resistance of α-phase aluminum bronzes.

Instead of modifying the composition in order to increase the cavitation erosion resistance, several studies aimed at improving the behavior at the interface between the cavitation-related phenomena and the base material. Surface engineering can provide adequate solutions to increase the cavitation erosion resistance via the deposition of hard layers attached to the base material. Ceramic coatings, like those based on alumina, titania, zirconia, and chromium oxide are known to provide improved properties of the base material, like the sliding wear and corrosion resistance. On what concerns the cavitation erosion of such ceramics, a significant difference was reported by Pedzicha *et al.* [10], with the alumina degraded by removing whole grains from the surface while the degradation of zirconia proceeded locally, along ribbon-like paths of removed grains. On metals, the ceramic coating is usually deposited by thermal spraying. Matikainen *et al.* [11] compared the effect of plasma and high-velocity oxygen-fuel (HVOF) spray processes of Al_2_O_3_ and Al_2_O_3_-13TiO_2_ powders on the erosion and cavitation erosion wear properties and observed that the highest wear-resistance in all tests resulted for HVOF spraying from fused and crushed powders.

Although the approach, involving the deposition of a new layer on the surface of the bronzes used for naval propellers, provides a flexible engineering solution, as shown by Gabriel *et al.* [12] not all the experimental tests provide a good mechanical and metallurgical compatibility between the layer and the substrate, and other even lead to a degradation of the surface layer or the substrate, with unwanted consequences concerning the sensitivity to brittle fracture. Such is the case for the experiments performed by Barik *et al.* [13] on thermally-sprayed nickel-aluminum bronze where the porosity introduced during the thermal spraying is detrimental to the corrosion protection offered by the thermal coating.

Yilbas *et al.* [14] showed that the use of laser treatments is efficient in improving the surface properties, including the cavitation erosion, and, while mostly used for steels it has also been considered for copper-based alloys, with positive results concerning the cavitation erosion resistance. Depending on the goals it can be used for surface heat or thermochemical treatments. Tang *et al.* [15] improved the cavitation erosion of copper-based propeller alloys by surface melting, Kac *et al.* [16] used the laser for alloying, while Yang *et al.* [17] used laser cladding of a Ni-base alloy on manganese bronze. Yilbas *et al.* [18] further used the laser for re-melting inhomogeneous layers that have been previously deposited on the surface.

The current work aims on improving the cavitation erosion resistance by modifying the properties of the surface via the formation of a new alloy on the surface of complex bronze-alloys by combining plasma-spraying with laser-beam re-melting of the deposited layer and a small fraction of the substrate. This way, the areas subjected to cavitation erosion will have improved metallurgical bonding to the substrate, thus providing an improved mechanical compatibility of the surface-layer-substrate system. The technique proposed in this work can be considered for the repair of large components; for example, turbine blades for hydroelectric power plants, for which the replacement costs would be extremely high.

## 2. Materials and Methods

The base material selected for the tests is a copper-based CuAl10Ni5Fe2.5Mn1 alloy, a standardized copper-aluminum alloy with iron and nickel (AMPCO 45 according to AMS 4640 (Aerospace Material Specifications), ASTM B.150 (American Society for Testing and Materials)), widely used and commercially available in various forms, including as forged or cast parts. Its chemical composition, according to EN is shown in Table 1.

Cavitation samples with a diameter of 15.8 mm were manufactured according to ASTM, G32-10 and cermet powder-type METCO 410NS (Oerlikon, Winterthur, Switzerland), with particles in the range of 170 ± 11 µm, was deposited on the surface of the samples. The composition of the powder, according to the manufacturer is detailed in Table 2.

The plasma spraying was made in a METCO SULZER robotized equipment (Oerlikon), using the following parameters: Ar + 6%H_2_ plasmagen gas at 9 bar pressure; Ar at 4 bar pressure as a transport gas, powder flow 63 g/min, 80–85 V voltage, and 550–600 A current intensity. The re-melting of the deposited layer was made with a Trumpf HL 124 P LCU Nd-YAG laser equipment (TRUMPF GmbH + Co. KG, Ditzingen, Germany), for several pulse powers (2200, 2400, and 2600 W), a pulse duration of 5 ms at 10 Hz frequency, with a speed of 4.07 mm/s.

The cavitation erosion experiments were made using a piezoceramic equipment manufactured according to ASTM G32–2010 standard, with a maximum of 50 μm vibration amplitude, 20,000 ± 2% Hz at a temperature of 22 ± 1 °C for the distilled water, used as cavitation fluid. The samples were attached to the end of the ultrasonic horn and subjected to intensive cavitation for up to 165 min. In order to assess the progress of the erosion on the surface of the sample the cavitation test was systematically interrupted at regulated time intervals (5 min until 30 min exposure and 15 min. after that). The samples were then cleaned in an ultrasonic bath and weighted. The mass loss was converted into volume loss and the results were analyzed and compared as mean depth of erosion (MDE) and mean depth of erosion rate (MDER).

In order to assess the accuracy of the cavitation erosion experiments, the dispersion band was re-plotted and the maximum and minimum values of the cumulative mean depth of erosion at the end of the test (after 165 min) were determined based on the regression curves and the standard deviation (s*_xy_*). A polynomial regression curve was used to fit the data, with the dispersion and the standard deviation further determined in order to plot the dispersion band.

The structural analysis of the plasma deposited and of the laser re-melted layer was made by using conventional metallographic investigation. The phases were identified by X-ray diffraction, using Cu Kα at 40 kV and 35 mA, while the Vickers hardness measurements (HV 0.2) provided additional information concerning the microstructural changes. The roughness of the samples was examined with a Mitutoyo SV-C3200 surface roughness equipment (Mitutoyo Corporation, Takatsu-ku, Japan). The topography of the surface prior to and after the cavitation experiments was analyzed in a TESCAN Vega 3 LM scanning electron microscope (SEM) (TESCAN Brno, s.r.o., Brno, Czech Republic‎), equipped with a Bruker Quantax 200 Energy Dispersive X-ray Spectroscopy (EDX) system with a Peltier-cooled XFlash 410M silicon drift detector (Bruker, Billerica, MA, USA).

## 3. Results and Discussion

**Figure 1:**
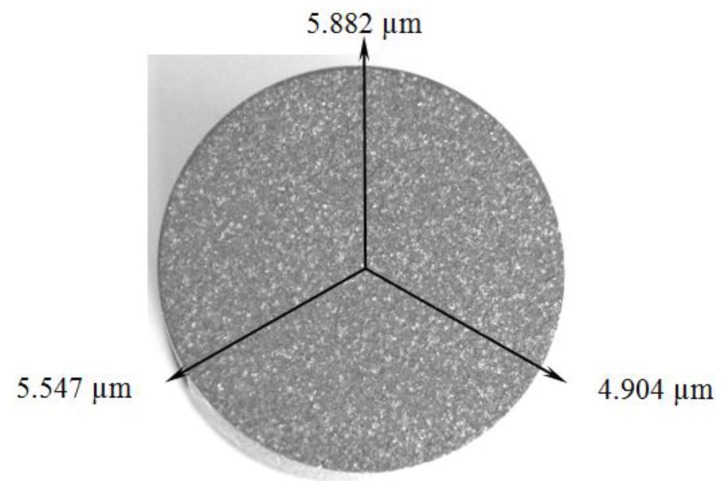
Macroscopic image of the Al_2_O_3_·30(Ni_20_Al) plasma deposited layer on the surface of CuAl10Ni5Fe2.5Mn1 sample (15.8 mm diameter) with the measured Rz data. Average Rz: 0.063 μm for the polished base material; and 5.445 μm for the plasma deposited layer.

**Figure 2:**
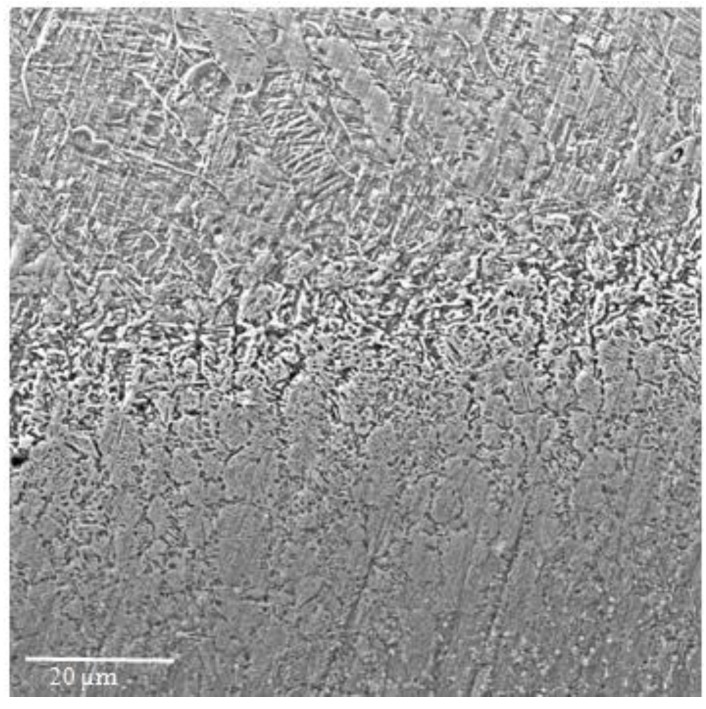
Details of the interface between the base materials and the plasma-deposited layer.

The results are detailed concerning several aspects, *i.e.*, the characterization of the used powder, the resulting surface layer after the plasma deposition, and the effects of the pulsed laser re-melting, before and after the cavitation erosion process. An 80–100 μm thick deposited layer with a uniform distribution of the un-melted particles was obtained following the plasma coating process (Figure 1). The analysis of the resulting interface, based on the microstructure shown in Figure 2, indicates a good connection between CuAl10Ni5Fe2.5Mn1 base material and the Al_2_O_3_ plasma deposited layer.

**Figure 3:**
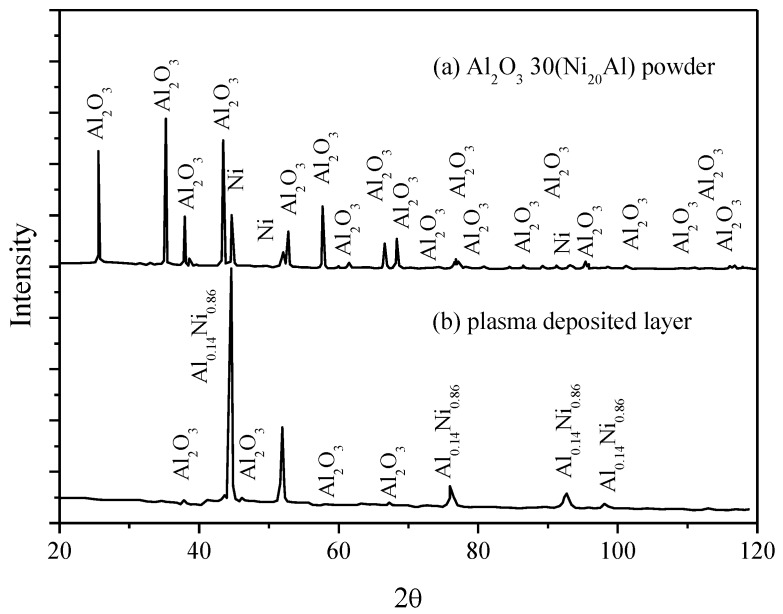
Comparative X-ray diffraction spectra of the Al_2_O_3_·30(Ni_20_Al) powder and of the resulting plasma-deposited layer on the surface of the CuAl10Ni5Fe2.5Mn1 sample. (**a**) Diffraction spectrum for Al_2_O_3_ 30(Ni_20_Al) powder; (**b**) Diffraction spectrum for plasma deposited layer

The comparative study of the X-ray diffraction patterns for the alumina powder and the deposited layer, in Figure 3, confirms on one side the presence in the powder used for the plasma deposition of the aluminum oxide, with excellent wear and temperature resistance, beside a small proportion of Ni (Figure 3a) and, on the other side, a Ni_0.86_Al_0.14_ phase dispersed in the matrix (Figure 3b). The high temperature developed during the plasma deposition (≈16,000 °C) favors the formation of layers with a reduced porosity (in the range of 2%–5%), out of powder materials with high fusion temperatures, such as oxides and refractory materials. Although Ar was used as a protective inert gas, the presence of a small quantity of oxygen in the deposition chamber is susceptible to oxidation.

**Figure 4:**
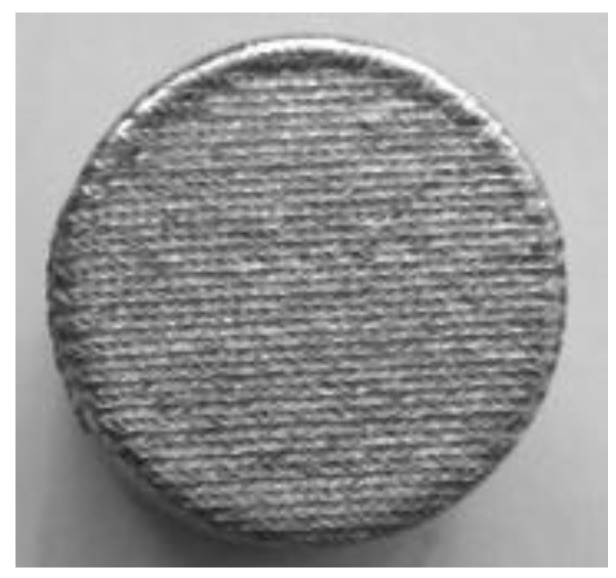
Macroscopic details of the surface of the laser re-melted sample prior to the cavitation erosion experiments (15.8 mm sample diameter).

The laser re-melting of the deposited layer and of the surface of the base material lead to a fine woven-like surface structure (Figure 4), regardless of the technological parameters used in the experiments.

This type of structure is especially difficult to clean during the cavitation experiments and plays a role in the mechanism of the erosion damage, especially during the first minutes of the cavitation attack. The composition analysis revealed an increase of Al (up to 35.32 wt %), Ni (up to 5.7 wt %), and Fe (up to 4.1 wt %).

**Figure 5:**
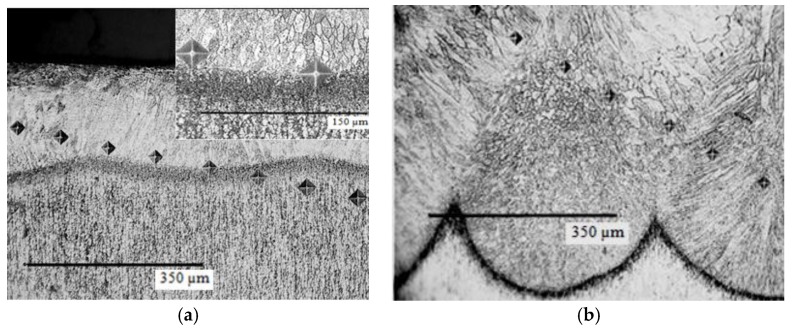
Cross-sectional microscopic details of the re-melted layer-substrate interface for different laser pulse powers. (**a**) Microstructure of the sample re-melted with 2200 W laser pulse power; and (**b**) microstructure of the sample re-melted with 2600 W laser pulse power.

The cross-sectional microstructure of the laser re-melted samples (Figure 5) reflects the advantage of melting with a high-power source that minimizes the width of the heat affected zone and allows a high heating rate. During the cooling phase, a dissipation of the heat occurs through the substrate material that acts as thermal absorbent. The melted zone front reaches the solidification temperature first in areas contacted to the surface, where the solid grains of the base material are present. The solidification of the melt starts there in a columnar way (Figure 5). In a similar way, the growth of the epitaxial grains from the melt can be explained (Figure 5b). The high cooling rate associated with a rapid solidification promotes a fine microstructure in the deposited layer-substrate interface that favors improved mechanical properties. Although the metallographic structure of Al bronze in controlled by the Al content, the supplementary alloying with Ni, Fe, and Mn, further preserves an α + β bi-phase microstructure (Figure 5b). The α phase (a solid solution of Al dissolved in Cu) confers excellent capacity of hardening by cold plastic deformation as well as good ductility and toughness, while the β phase (a solid solution based on Cu_3_Al electronic compound), preserves good high temperature deformation properties and high hardness.

**Figure 6:**
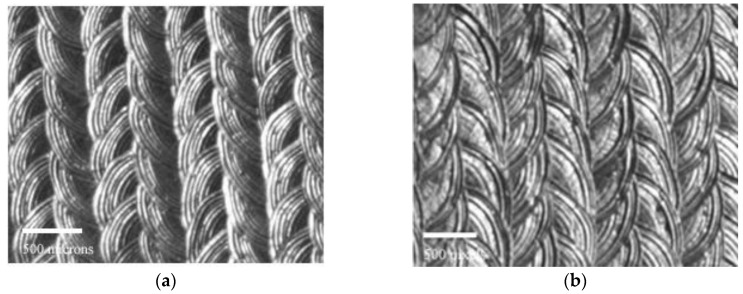

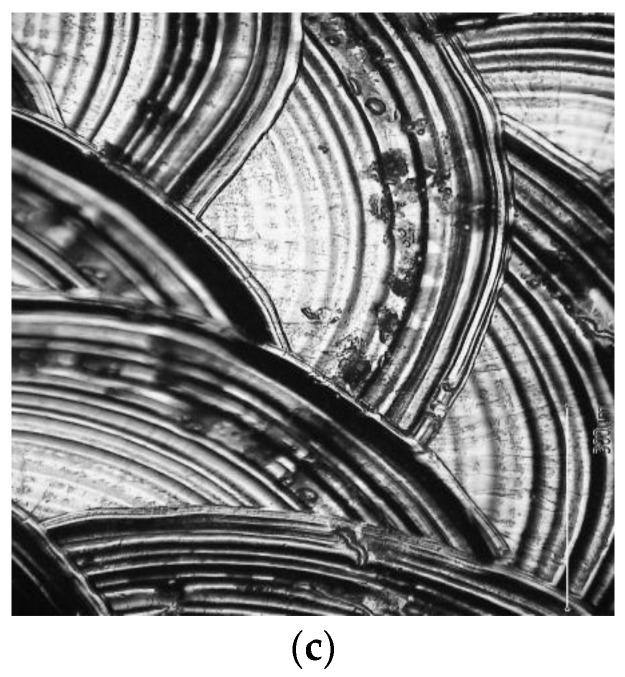
Surface features of the re-melted layers for different laser pulse power. (**a**) Surface topography of the sample re-melted with 2200 W laser pulse power; (**b**) Surface topography of the sample re-melted with 2600 W laser pulse power; and (**c**) magnified image of the surface topography of the sample re-melted with 2600 W laser pulse power.

Overall, the microstructure is composed of 55%–65% α phase and β, the rest (determined by quantitative metallography). Thus, a good combination between mechanical and plastic properties is expected. This is controlled by the contents of Al, Ni, Fe, Mn, and by the cooling conditions. Iron provides a fine grain microstructure and, along with Ni and Mn, additionally contributes to the improved mechanical characteristics. In the samples, a good mechanical compatibility is obtained, since there is a metallurgical link between the laser re-melted layer and the substrate. No major defects, such as micropores or voids, are identified at the layer-substrate interface and the heat affected zone is limited, due to the localized heating (also proved by cross-sectional hardness measurements, discussed further on). EDX data recorded for the re-melted surface (with the topography exemplified in Figure 6), further confirms the presence of a new alloy in the surface layer, with a significantly higher amount of Al (about 34% compared to 10%) and a slightly higher amount of Ni (about 5.60% compared to 5%) content than in the substrate.

**Figure 7:**
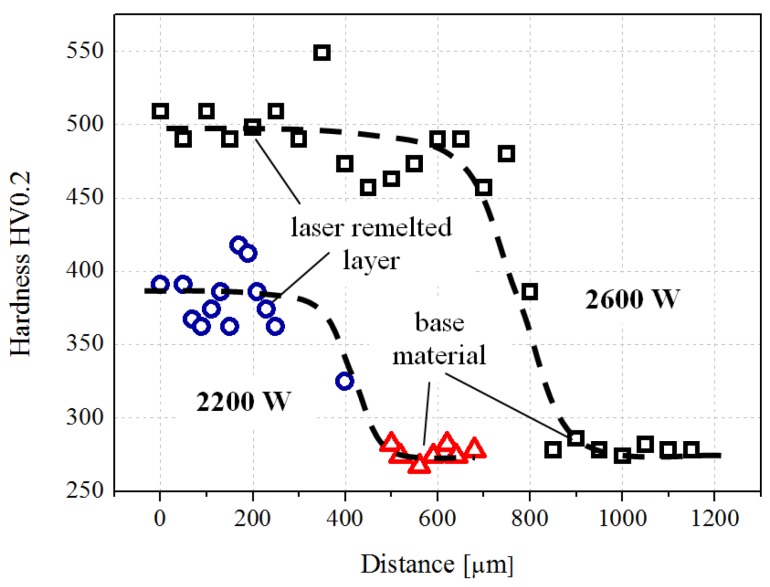
Hardness difference between the surface layer and the base materials (for 2200 W pulse power) and hardness profile across the interface (for 2600 W pulse power).

The changes in the chemical composition and the microstructure that occurred in the surface layer lead to a significant increase of the HV 0.2 microhardness, compared to the base material (Figure 7). The increase of the laser power from 2200 to 2600 W not only leads to an increase in the thickness of the newly-developed composite layer (from about 200 μm to about 800 μm) but also to an increase in hardness from 350–400 to 400–500 HV 0.2). The increase of the laser power is associated with the increase of the layer thickness and of the microhardness due to the pronounced dissolution of the powder particles in the solution and a supplementary alloying of the metallic matrix of the base material. Compared to the base materials with a microhardness in the range of 275–285 HV 0.2, the surface microhardness is increased by 43%–78%, depending on the laser re-melting power.

The statistical data detailed in Table 3 for the cavitation erosion experiments determined from the dispersion bands for 2200 and 2600 W pulse power show a similar behavior for the samples tested, with minor differences related to the complexity of the hydrodynamic cavitation process and its erosion mechanism.

The low value for the standard error confirms the validity of the experimental procedure in which the erosion was produced using the piezoceramic vibrating equipment is uniformly produced across the surface of the sample.

**Figure 8:**
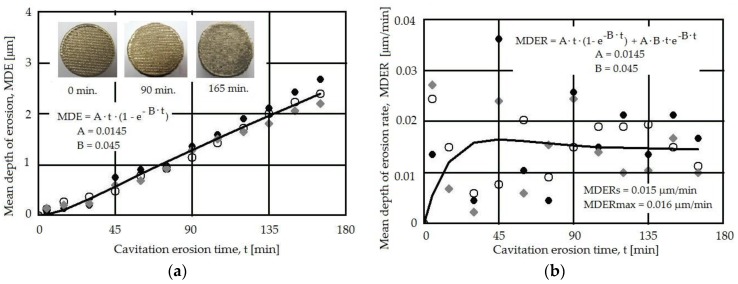
Cavitation erosion curves for the 2200 W laser pulse power. (**a**) Mean depth of erosion (MDE)(*t*); (**b**) Mean depth of erosion rate (MDER)(*t*).

**Figure 9:**
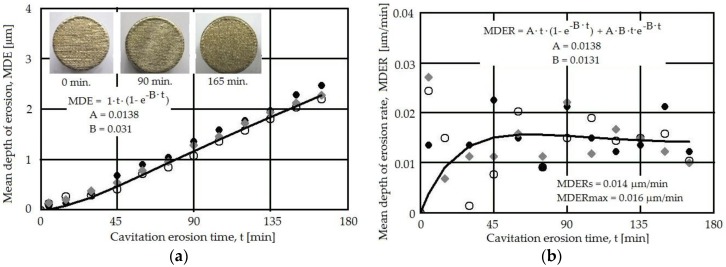
Cavitation erosion curves for the 2600 W laser pulse power. (**a**) MDE(*t*); and (**b**) MDER(*t*).

The values for the mean depth of erosion (MDE) and mean depth of erosion rate (MDER) were determined based on the mass loss determined by periodically weighting the samples [19]. Figure 8 and Figure 9 show the MDE and MDER *vs.* the exposure time for two powers used in the laser re-melting phase (2400 and 2600 W) and reflect the corresponding cavitation erosion behavior. The large dispersion of the data at the beginning of the cavitation erosion process (first 15 min) is due to the impossibility to fully eliminate the abrasive particles from the eroded surface and of the asperities peaks. The process further stabilizes for longer exposure times.

The surface shape plays a role in the cavitation erosion process, since the entrapment of air bubbles in the already-formed caverns is a factor in the evolution of the cavitation erosion process [2]. In our case, the woven-like pattern allows the attenuation of the impact pressure of the microjets and the shockwaves by the air captured between the surface protrusions, leading to a different behavior compared to a flat polished surface with a roughness Ra = 0.2 μm.

Both the MDE(*t*) and MDER(*t*) experimental curves reflect the cavitation erosion process well, with a dispersion of the experimental data compared to the fitting curves indicating a similar behavior for the two laser powers used. This can be explained by the fact that both laser regimes provide a similar effect in the surface, leading to high microhardness and a relatively homogenous distribution in this surface (also reflected by the microhardness data in Figure 7). The macrograph insets in Figure 8 and Figure 9, recorded at different exposure times, do not show evidence of major differences, mainly due to the already-patterned surface that resulted during the laser melting process. The stabilization of the MDER(*t*) and the similitude in the data dispersion in the same range of cavitation time, for both laser powers, indicate that the same erosion mechanism is present in both cases on what concerned the energy absorbed during the impact with the microjets and shock waves. The small difference in the experimental data points is related to the particular effects of the cavitation erosion energy absorbed during the plastic deformation, crack, or fracture processes and the material’s expulsion.

**Figure 10:**
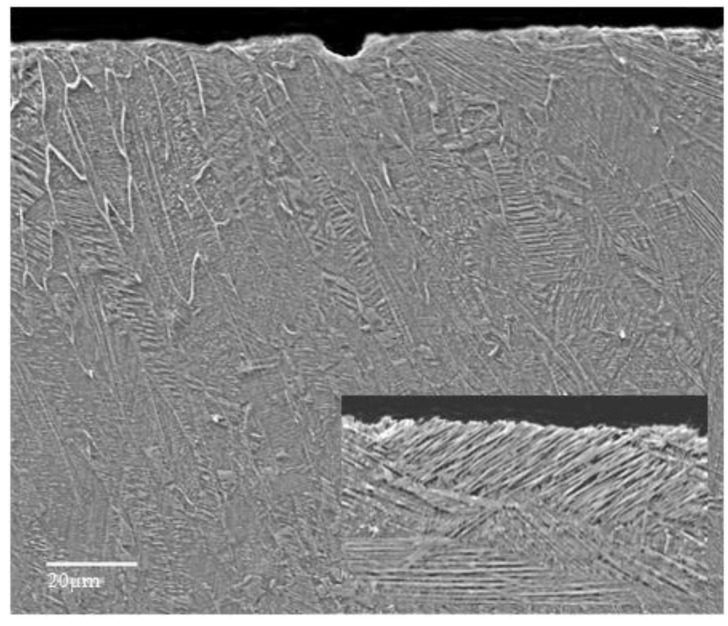
Cross-sectional details of the cavitation erosion effects. The inset shows the acicular substructure.

The analysis of the cross-sectional microstructure of the surface eroded by cavitation, exemplified in Figure 10, shows the presence of a columnar microstructure with acicular substructure oriented towards the thermal gradient. This is the result of a rapid cooling of the melted surface layer. Although the re-melted structure would favour the initiation of the cavitation erosion process at the interface of the columnar grains, a rather uniform deterioration of the surface is observed, with local pits that may appear in regions where chemical combinations of Al and Ni are present.

**Figure 11:**
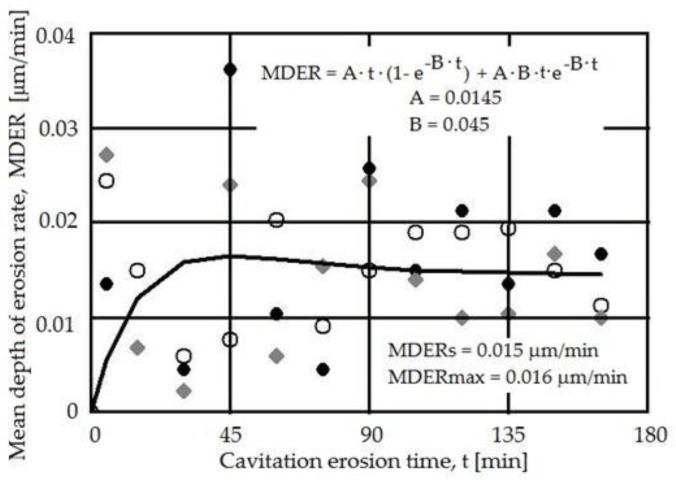
Comparative image of cavitation erosion resistance (reflected by the MDE *vs.* cavitation erosion time), indicating the improvement as a result of the alumina coating. The 2200 and 2600 W denote the parameters used for laser re-melting of the surface layer, following the Al_2_O_3_·30(Ni_20_Al) plasma spraying. AMPCO 45 is the base metal shown for comparison purposes.

In Figure 11 the results of the cavitation erosion experiments, reflected by the MDER(*t*), for the alumina-coated CuAl10Ni5Fe2.5Mn1, for two laser re-melting powers, are compared to stainless steel used in components for hydraulic equipment, as well as with AMPCO 45 alloy [20], usually used in heavy-duty high-loaded mechanical and corrosive applications (all samples were tested under the same conditions).

Based on the comparative results in Figure 11, the cavitation erosion resistance improves in the stabilized zone by up to 11 times, compared to the reference stainless steel and by about three times, compared with the naval bronze.

The similitude in the behavior of the two samples subjected to different laser power surface re-meltings is proven by the erosion rate in the same range (MDER_max_ = 0.016 μm/min, and minor differences between the erosion rate for both regimes). These values are characteristic for materials with good cavitation resistance, thus this technique can be considered for improving the behavior of ship propellers.

## 4. Conclusions

The cavitation erosion resistance of CuAl10Ni5Fe2.5Mn1 improved following plasma deposition of Al_2_O_3_·30(Ni_20_Al) powder and laser re-melting of the deposited layer. The mechanical characteristics of the surfaces, especially the hardness (from 250 to 420 HV 0.5), provide a high resistance to the cavitation erosion attack, up to six times compared to the one of the base material.

The surface woven-like pattern resulting from the laser re-melting and the microstructure of the deposited layer, consisting of a cubic Al_2_O_3_ matrix with dispersed Ni-based solid solution, play a positive role in the cavitation erosion resistance. The fine microstructure formed at the interface provides a good connection to the bronze substrate and can explain the resistance to plastic deformation and, implicitly, its uniform and smooth degradation during the cavitation erosion process.

A change in the laser re-melting power from 2200 to 2600 W is not associated with major changes, for both cases in this range the deposited and re-melted layer improves the cavitation erosion resistance by about 11 times compared to X_5_CrNi_18.9_ austenitic stainless steel and by about three times compared with the known Cu-Ni-Al bronze. A uniform deterioration of the surface resulted after the cavitation erosion, with local pits that may appear in regions where chemical combinations of Al and Ni are present.

Based on the experiments performed on CuAl10Ni5Fe2.5Mn1 with an Al_2_O_3_ plasma deposited layer and laser re-melted surface, it is concluded that the resulting high-resistance coating is a solution that can be considered for improving the cavitation erosion resistance of bronzes used for components operating in hydraulic equipment.

## Figures and Tables

**Table 1:** Chemical composition of AMPCO 45.

Element	Al	Ni	Fe	Mn	Other	Cu
**Content (wt %)**	10	5.00	2.5	1.00	≤0.5	81–81.5 as balance

**Table 2:** Chemical composition of the METCO 410NS.

Components	Ni_20_Al	TiO_2_	SiO_2_	Fe_2_O_3_	Other Chemical Elements	Al_2_O_3_
**Content (wt %)**	29–31	<3	<1.5	<0.7	<1	rest

**Table 3:** The values of the statistical parameters for the cavitation erosion experiments.

Laser Pulse Power	2200 W	2600 W
Mean depth of erosion after 165 min [μm]	2.391	2.262
Maximum value according to the regression curve [μm]	2.659	2.663
Minimum value according to the regression curve [μm]	2.123	1.861
Standard error [μm]	0.089	0.134
